# Functionally oriented music therapy (FMT) in the treatment of long-term musculoskeletal pain. A qualitative observational study

**DOI:** 10.1080/17482631.2025.2545674

**Published:** 2025-08-10

**Authors:** Alhusayn Alqarqani, Louise Eulau, Johanna Fritz, Lena Nordgren, Helena Lööf

**Affiliations:** a School of Healthcare and Social Welfare, Mälardalens University, Västerås, Sweden; b Department of Nursing Science, Sophiahemmet University, Stockholm, Sweden; c Department of Clinical Science and Education, Karolinska Institute, Stockholm, Sweden; d Centre for Clinical Research Sörmland/Uppsala University, Eskilstuna, Sweden; e Department of Public Health and Caring Sciences, Uppsala University, Uppsala, Sweden

**Keywords:** Functionally oriented music therapy, long-term musculoskeletal pain, observational study, person-centred care, qualitative study, treatment in long-term musculoskeletal pain

## Abstract

**Purpose:**

The aim was to interpret and describe functionally oriented music therapy (FMT) as a method in the treatment of long-term musculoskeletal pain in a region in the middle of Sweden. A first step of a larger project aiming to study the method’s effectiveness on participants pain and daily life qualitatively and quantitively

**Methods:**

The study was a qualitative observational study. Video observations were supplemented with semi-structured interviews with FMT therapists (*n* = 3) and participants with long-term musculoskeletal pain (*n* = 19). Collected data was analysed using thematic analysis.

**Results:**

Five main themes were identified in connection with the main core theme of “Person centred tailored treatment”. The video observations have shown no negative impacts during the sessions. Positive body language was observed (i.e. smiling, laughing) that’s in line with curiosity and joyfulness. This was also confirmed in the interviews.

**Conclusions:**

FMT holds promise as a therapeutic treatment for long-term musculoskeletal pain conditions. By utilizing music as a communication method alongside body language, individuals can experience pain relief, emotional regulation, and improved quality of life. Further research and clinical application of FMT can potentially enhance the overall care and well-being of individuals living with long-term musculoskeletal pain.

## Introduction

Pain is the most common reason why people seek primary care, and long-term musculoskeletal pain is also one of the most common reasons for long-term sick leave (Statens beredning för medicinsk och social utvärdering, 2010). In this study, the FMT method is studied in the context of patients with long-term musculoskeletal pain. With long-term musculoskeletal pain defined as pain that affects bones, muscles, ligaments, tendons, and even nerves that persist or recur for longer than 3 months and is associated with significant emotional distress or functional disability (El-Tallawy et al., 2021; Treede et al., 2019). Living with long-term pain can have a major impact on people’s social context (e.g., sense of self and self-identity, one’s experience of the body, social and work role function, and personal relationships with significant others, including family, friends and health professionals) which creates a struggle with legitimacy, credibility and personal integrity and the experience of being a burden on society (Biguet et al., 2016; Raja et al., 2020). In addition, it is described that there is a stigmatization of people with long-term musculoskeletal pain as the healthcare systems in place fail to meet their needs and expectations. Creating a feeling of stigmatization when they do not receive a diagnosis. It is also common for people living with long-term pain to have low confidence in their own ability for activities, which can contribute to the person withdrawing from activities with loneliness and isolation as a result (Biguet et al., 2016). The management of individuals with long-term pain conditions is complex (Steihaug et al., 2001). Even when patients with long-term pain share the same medical diagnosis, they do not form a homogeneous group, and their responses to treatment will vary (Turk et al., 1996). As several aspects affect the pain experience, such as intensity, quality, and duration and pain having diverse pathophysiologic mechanisms and meanings (Raja et al., 2020). Many times, a multi-modal support with a palette of varied support efforts is needed for a new orientation in life such as multi-professional pain rehabilitation programmes. The program follows evidence-based principles and consists of cognitive behavioural therapy, education, group discussion, physical exercise, body awareness therapy, mindfulness and occupational training such as pacing and goal setting (Biguet et al., 2016). Meaning that there are many ways to treat and reduce long-term pain, aches and the suffering that is often associated with the pain (Biguet et al., 2016; Raja et al., 2020). One of those ways is personalized physical movement and exercise as they form an important part of rehabilitation in pain and show how self-efficacy for activity is important for recovery (Söderlund & Asenlöf, 2010). Another form of reducing long-term pain is various cultural activities that use music to reduce pain such as cultural ceremonies, celebrations or rituals which may provide social support networks and coping mechanisms for individuals experiencing pain (Okolo et al., 2024).

Research has shown that listening to music and music therapy can increase people’s well-being and provide distraction so that pain and illness are alleviated (Fancourt & Finn, 2019; Juslin, 2019; Tervaniemi et al., 2021). Studies have shown that music and musical activity relate to feelings of control, memories, and associations so that experiences of pain and discomfort are distracted and reduced, whereby positive emotions and increased wellbeing are experienced (Juslin, 2019; Tervaniemi et al., 2021). Several models and approaches describe major traditions in music therapy globally for example, Nordoff-Robbins, GIM, or community music therapy (Edwards, 2015). Like nursing, music therapy continues to evolve, influenced by fields such as occupational therapy, psychology, psychotherapy, special education, music education, music psychology, anthropology, and medicine. This has led to diverse and broad music therapy theory and practice both internationally and in Sweden (Paulander, 2011; Trondalen & Ole Bonde, 2012), creating a dynamic landscape for ongoing research, development, and innovation within the field of music therapy. There are several methods and techniques in music therapy where therapists and “clients” (a term used by music therapists) people engage in musical experiences (Trondalen & Ole Bonde, 2012). One of the most established music therapy methods in Sweden is Functionally Oriented Music Therapy (FMT). This body-based and music therapeutic method is used for health-promoting purposes (Hjelm, 2005; Jonsson, 2014; Paulander, 2011). However, due to the lack of detailed descriptions in the main sources (Hjelm, 2005), there is room for interpretation of the method and its functionality and effects.

FMT is based on a neuromuscular sensorimotor basis and focuses on specific basic human functions: body control and movement patterns, perception, cognition, and emotion (Hjelm, 2005; Jonsson, 2014). It was initially developed by music teacher Lasse Hjelm with his expertise in physiotherapy and occupational therapy among other things (Hjelm, 2005) for children with neuromuscular disabilities of varying degrees in Sweden. Hjelm collaborated closely with physicians, educators, psychologists, and other professionals from fields integral to children’s development (FMT-behandlingscentrum (u.å.), n.d.). Together, they tailored individualized training programs with the help of e.g., drums, cymbals, various custom-made drumsticks, flutes, chairs, balance balls and balance cushions for each child or “adept,” a term he borrowed from educational theory as he drew inspiration from Jean Piaget’s theory (Mcleod, 2009). This individualized approach became the foundation for the FMT method as a person-centred tailored treatment which is taught and used by Hjelm’s students up to this day. The FMT method focuses on providing a room, or a space, where structured sensorimotor experiences (playing instruments, combining sound to movement helping them to connect sensory experiences with motor actions) can take place in a safe and fun environment free of demands and judgement as well as any sensory distractions such as bright lights, loud noises, too many visible instruments … etc. This space is called the FMT room. The specially composed music used in FMT consists of various combinations of melodic lines, rhythms, and harmonies in short phrases called “codes” composed by Hjelm himself. He decided to avoid all commercial music and instead compose his own, original melodies that would not conjure up any associations that could affect the adept’s mood and/or focus. These codes are linked together through different instrument combinations and movement patterns. Each code uniquely combines motor movements and cognitive activity (Jonsson, 2014).

The music therapy sessions are structured around rituals that can be categorized into three phases: an entering phase, a current (transcendence) phase, and an exit phase (Paulander, 2011):

- **Entry Phase**: This is the initial phase where the music therapist establishes a rapport with the client, sets the goals and objectives of the therapy, and introduces the client to the music therapy process. This phase starts with harmonic simplicity where the adept only listens to the introductory tune played by the therapist to signal the start of the session.
- **Current Phase**: This phase involves the active music therapy process where the adept engages in music-making activities, by playing on the drums while the therapist plays on the piano. The music therapist observes and responds to the client’s musical expressions.
- **Exit Phase**: This final phase involves the client’s transition from the music therapy process of making music to the end of session section where they only listen to music and relax. This phase ends as well with harmonic simplicity where the adept only listens to the therapist play the final tune indicating the end of the session.

While these rituals are typically predetermined and guided by the principles of music therapy, some variations may still be observed. This means that the participants in FMT play selected music during the entering- and exit-phases and then play a little bit more varied music during the ongoing phase. Furthermore, the FMT method follows a non-verbal, body-based communication method. It is of importance for the FMT-therapist to be aware and responsive to the body signals of the participants. Through participation in FMT, the conditions for movement with a balanced body awareness should be created to ensure participants comfort and offer a painless session (Paulander, 2011). Furthermore, body awareness includes an ability to pay attention to changes in the body’s signals, for example for sleep/wake rhythm, the energy balance in the body, as well as attention to ill health (Mehling et al., 2009). A Swedish pilot study suggests that FMT improved the quality of life in chronic stroke survivors and on patients with Parkinson’s disease (Rosin et al., 2015). Since music and physical movement are shown to be beneficial in long-term pain treatment (Ambrose & Golightly, 2015; Garza-Villarreal et al., 2017), FMT combining both could potentially lead to an improved outcome in the recovery process. To our knowledge, however, there are no studies that have evaluated FMT in long-term musculoskeletal pain. The lack of research in FMT as a treatment for long-term musculoskeletal pain leaves a knowledge gap regarding any benefits or harms FMT could possibly hold regarding treating long-term musculoskeletal pain. This study aims to interpret and describe FMT as a method in the treatment of long-term musculoskeletal pain in a region in the middle of Sweden.

## Methods

Critical realism was the methodological approach for this study as it is underpinned by the belief that reality exists in multiple layers. It has gained increasing acceptance across various fields, particularly in research that recognize the complexities of the social environment and aim to provide answers to real problems (Mingers, 2010; Syed et al., 2010). The FMT treatment is currently offered as part of a care agreement in at least one region in Sweden, targeting a large and diverse group of patients with long-term musculoskeletal pain. Given the limited research on FMT in practice, and the heterogeneity of this patient group, the research team aimed to explore the treatment from multiple perspectives. To support this aim, the team included researchers from different professional backgrounds—medicine, nursing, physiotherapy, and musicology—providing a variety of viewpoints. Realistic approaches rooted in critical realism are well-suited to the complex nature of healthcare practices. It enables researchers to transcend the cultural barriers present in these other perspectives (Williams et al., 2017).

### Design

**Figure 1.**
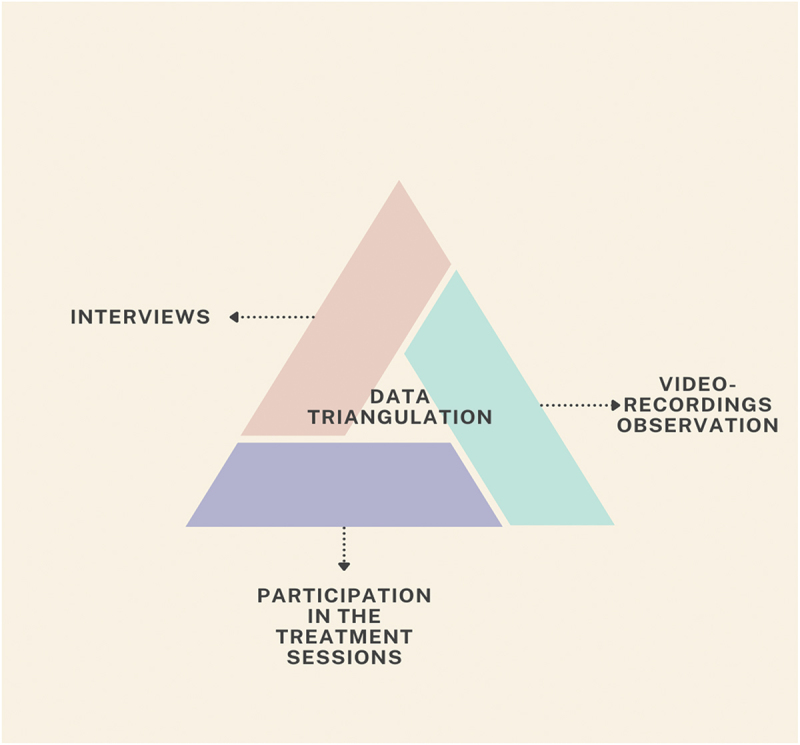
Data triangulation.

This study employed a qualitative mixed-methods design using data collection triangulation, involving multiple methods of data collection about the same phenomenon—FMT as a method (Carter et al., 2014). This approach enabled a richer and more nuanced understanding of the complexity of FMT, beyond individual experiences. While drawing on the perspectives of both participants and music therapists, the focus was on interpreting and describing the characteristics, components, and underlying mechanisms of FMT as a therapeutic method. The triangulated data collection consisted of qualitative data collected through interviews with participants and music therapists, video recordings of treatment sessions, and participation in 3 FMT sessions by researcher AA (Figure 1).

### Participants

Both FMT therapists and people with long-term musculoskeletal pain were recruited for this study. For ease of reference, in the following text, the FMT therapists will be referred to as “therapist/s” and people with long-term musculoskeletal pain as “participant/s”. Neither the therapists nor the participants have any had any form of connection or relationship with anyone from the research team prior to the study commencement.

All therapists (*n* = 3) were recruited from a region in Sweden where FMT is offered as a treatment for long-term musculoskeletal pain conditions as part of a care agreement between the region’s health board and the therapists in the region. There is no personal connection between the therapists and the research team.

The participants (*n* = 19) included in this study had moderate to severe levels of long-term musculoskeletal pain measured by VAS (visual analogue scale) (Statens beredning för medicinsk och social utvärdering, 2010). A purposeful sampling approach was used (Palinkas et al., 2015). Participants were recruited based on them receiving FMT as a treatment and fitting the selection and exclusion criteria. Furthermore, the therapists were encouraged to try and recruit a diverse group of participants if feasible due to the time and work limit. There is no personal connection between the therapists and the research team. Demographic characteristics of the participants are presented in Table I.

**Table I.** Demographic characteristics of the participants (*N* = 19) and therapists’ length of practice (*N* = 3).

Participants	
Gender	
Male	2
Female	17
Mean age in years (min – max)	55 (35–78)
Mean pain level on VAS* (min – max)	6.8 (5–10)
**Therapists**	
Mean length of practice in years (min – max)	18.6 (3–31)

*VAS (Visual Analog Scale) range = 0–10

The participants were receiving FMT as a treatment by the recruited therapists and informed about the study by the therapists and given an information letter about the study and had freedom of choice on whether to participate in the study or not.

All video recordings and interview recordings were conducted with both verbal and written consent from the participants with long-term musculoskeletal pain and therapists.

### Eligibility criteria for participants with long-term musculoskeletal pain

- People with long-term musculoskeletal pain that occurred for at least three months.
- People over 18 years of age.
- People able to understand and speak Swedish or English.

### Exclusion criteria for participants with long-term musculoskeletal pain

- Severe hearing loss, visual impairment or cognitive impairment.
- Pain conditions caused by malignancy, severe spinal pathology and neurological disease that severely limit the ability to perform activities.

### Setting

The treatment sessions took place in either in a room in the FMT centre (*n* = 6) or in a room at one of the several primary care centres in the region in collaboration with the FMT centre (*n* = 13). The room where the session takes place is quiet with no noise and only one therapist and one participant with long-term musculoskeletal pain in the room alongside the instruments used for the session. The door is kept open in a welcoming manner so participants can just walk in instantly to their appointment. As the participants with long-term musculoskeletal pain enter the room, they immediately find a chair chosen for their best comfort to sit on. After the participant enters the room and sits down, the therapist closes the door to block any outside noises and/or distractions. After the participant sits and door is closed the session starts immediately in a comfortable quiet manner.

### Data collection

The data collection took place from September 2023 to March 2024, during which 19 people with long-term musculoskeletal pain and 3 therapists were interviewed, supported by 19 video-recordings of the treatment sessions. Video recordings of the FMT treatment sessions were made by the therapists using a stationary camera placed in the corner of the room behind the therapist and facing the participant. Only the therapist and the participant were present during the recording. Each session lasted approximately 20 minutes. Interviews were conducted 2 to 4 days after the session and lasted approximately 40 minutes on average. Participants had the option to take a break and continue on a later time or date if needed in order not to overwhelm them. This timing considered their long-term musculoskeletal pain condition and their need for rest. Furthermore, it allowed enough time to schedule the interview time.

Semi-structured interviews (Mason, 2002) were done and recorded by researchers HL and JF (see Tables AI and AII in Appendix for interview guide). The interviews were conducted via digital communication tools (Zoom or Teams) as a choice and via telephone in case of inaccessibility to digital options and for the participants’ comfort. The interviews were audio recorded on a Dictaphone. 17 interviews were done via telephone and 2 via digital video calling. Interviews with the therapists were done in-person (face-to-face). Interviews with the participants began with an opening question “When I say music, what do you think of?” Then, follow-up questions were asked such as, “‘Can you tell me more?’” and “‘How do you mean?’” These questions were designed to support the participants in their reflection and to help them to express variations in their experiences. Furthermore, an interview guide was designed to explore the meaning of FMT from the perspective of both the participants and the therapists. While the interviews provided insight into participants’ experiences, the overarching aim was to contribute to a broader understanding of FMT as a therapeutic method. Participants experience regarding the effectiveness of FMT on their daily life and pain will be further studied and reported in upcoming studies and papers as this current paper is part of a larger research project. The interviews with the therapists began with an opening question “When I say FMT, what do you think of?” Then, follow-up questions were asked such as, “Could you tell us a little bit about how FMT fit as a treatment method?” “How to make it?” And “Can you give us examples of some of the instruments and tools you use during a treatment session?” These questions were designed to support the therapists in their reflections and to help them cover more details which might be missed otherwise. By focusing on both the practical and conceptual aspects of FMT, these questions aimed to capture how the method is understood and applied in clinical practice, contributing to a deeper interpretation of FMT as a therapeutic approach.

Researcher AA personally underwent 3 FMT sessions after the completion of the data collection to re-enact the experience of a participant undergoing the FMT treatment. This was done to collect additional data about the setting and the FMT experience and how it feels. Where researcher AA was welcomed and treated as a participant attending FMT and went through 3 sessions once a week for 3 weeks. This provided deeper insight into the treatment method and approach and allowed for a more comprehensive understanding of the video-recordings and the descriptions given by the therapists and participants.

### Data analysis

To interpret and describe FMT as a method in the treatment of long-term pain, the analysis concentrated on formulating themes. The themes were based on the participants’ and therapists’ interview transcripts, the video-recorded sessions and participation in 3 FMT sessions by researcher AA. This approach offered opportunities to go deeper into the subject matter. Thematic analysis was deemed the most suitable method to analyse the data because it is a flexible method that allows for a variety of ontological and epistemological viewpoints (Braun & Clarke, 2006). Thematic analysis represents a systematic framework to code qualitative data to identify patterns across the data (Braun & Clarke, 2014). Furthermore, a qualitative descriptive approach was used to gather rich descriptions about the treatment sessions, which little is known about. Within the analysis process, the researchers strived to remain closely aligned with the participants expressed experiences (Bradshaw et al., 2017). In this process, the experience was described from multiple viewpoints—including those of the participants, the therapists, and the researchers’ own observations—in order to construct a detailed report of the treatment sessions. Braun and Clarke (Braun & Clarke, 2006) outline a series of phases through which researchers must pass to produce a thematic analysis. Steps involved in thematic analysis include familiarizing oneself with the data, generating initial codes, searching, reviewing and finally, defining themes (Braun & Clarke, 2014).

Firstly, all the data collected during the interviews were transcribed manually in Microsoft Word by researcher AA and validated by researcher HL. After the initial transcription the transcriptions were carefully re-read while listening to the interviews again to ensure nothing was skipped; identifying details, such as full names, countries, cities, and workplaces, were not collected but were hidden in case mentioned and not used. After this, the texts were reviewed twice more—after the transcription of one entry was completed and after the entire transcribing phase was completed. Secondly, after the 22 interviews were transcribed (19 participants and 3 therapists) and reading the transcripts multiple times and writing down initial thoughts and ideas, codes were generated by dividing the transcripts into smaller meaningful units and coding for as many potential patterns as possible. Thirdly, searching for themes by listing the codes and sorting them into potential theme sections. Fourthly, reviewing the themes and refining them by comparing them to the transcripts and conceive a satisfactory agreed upon thematic map among the research group. Fifthly, defining and naming the themes and identifying the main core meanings of the themes and naming them accordingly. The final themes uncovered were (Progressive encouraging movement, Continuous learning journey, Joyful, Distraction and focus in a stress-free environment, and Non-verbal communication) which all branch out from the core, main theme “Person centred tailored treatment”. Lastly, producing the report of the data by using clear, meaningful data extracts.

The video observation analysis took the same steps. Starting with familiarizing oneself with the data as researcher, AA watched the participants videos multiple times before participating in the sessions himself, and multiple times again after participating in the sessions to gain a deeper insight on FMT. After the 19 videos were observed fully, initial thoughts and ideas covering the essence of what was seen in the video recordings were written down by researcher AA and validated by the research team. Codes were generated by coding for as many potential patterns as possible observed in the videos which afterwards were used to search for categories shared across all the videos. The final steps included reviewing the categories and refining them to reach a final set of categories seen across all the videos. Five categories were developed across all the videos (Situation, Interaction, Pain, Music, Movement) to write an interpretation of the videos and provide a comprehensive description of the setting and what happens in the sessions. Finally, a descriptive report of the treatment session was written based on the gathered information from the interviews and the video observations.

### Ethical considerations

Research ethics principles of confidentiality, informed consent and voluntariness was described in an information letter provided when requesting participation and video recording of FMT sessions. This study was approved by The Swedish Ethical Review Authority under approval number 2023–03095–01. The participants were repeatedly assured that all information will be treated confidentially, and that no individual information will be disclosed to the public. All data is saved and stored following General Data Protection Regulation (GDPR) and the Swedish ethical review authority guides. Contact information of the research team was given to the participants that agreed to participate in the study and are free to contact the researchers with any questions they might have. The participants were able to participate in the treatment even though they declined to participate in the study.

## Results

**Table:** 

**Text box 1. Descriptive report**. In FMT, music and movement are combined for health-promoting purposes (Hjelm, 2005), which can be assumed to lead to experiences of well-being and recovery. Regarding the target group (participants with long-term musculoskeletal pain conditions), the setting of the session is prepared in accordance with their pain. Specific chairs are used based on the participants’ comfort (height adjusted, cushions added to sit on or to relax their back on). The sessions are observed to start with the traditional initial entering phase where the music therapist plays an introductory tune to establish that the session has started, afterwards begins the ongoing phase where the drums and cymbals are positioned in front of the participant to start playing on. Depending on how many sessions the participant had had before and how familiar they are with the method, the number of drums and cymbals can vary. For first timers, a snare drum is placed in front of them. They’re given a pair of drumsticks before the therapist starts playing on the piano while they are expected to play on the drum. At the end of session, the therapist concludes the session with an exit tune that’s always played at the end as part of the exit phase. While simultaneously giving the participant an anti-stress ball to hold. The anti-stress ball and exit tune is what hints to the participants that the session is ending (Juslin, 2019). In case the participant doesn’t start playing, the therapist stops playing on the piano to hint that it is time for the participant to join in. Once the participant strikes the drum once, they notice that the therapist continues playing. In the initial sessions, participants often feel unsure and look to the therapist for guidance. The therapist maintains eye contact and nods when the participant strikes the drum, offering reassurance and encouragement. Participants try to figure out the pattern of playing by observing the therapist and listening to the music. The participants try to hit the drums and cymbals in different orders depending on the current set. In situations where the participants are not able to figure out where and how to start completely, more hints are given by the therapist. As the therapist approaches the participant to hand them the drumsticks, once the participant grabs the drumsticks, the therapist guides their hands by pulling the drumsticks to the drum or cymbal where they should start. The therapist would hold the drumstick down on the drum where the starting point is. In its essence, participants learn by trial and error. Now, as the sessions progress and the participants understand what to do better, more and more drums and cymbals are added for more, better movement simulation. It starts with moving the instruments further away to promote more reach and extension movement. In certain sets, the instruments are put further to the side and to the back to promote more body core and rotational movement. Drumsticks as well are constantly changed throughout the session as FMT also focuses and pays attention to wrist movement and grip strength. Drumsticks can also be changed simply after observing that the participant is uncomfortable using the current pair of drumsticks. The different drumsticks focus on different hand/wrist functions and movement and are also picked based on what the participants need, and which ones would be best for their condition. With more drums and cymbals used, as well as the different drumsticks used throughout the session, more attention and focus is needed by the participants to follow through the set which is a core part of FMT as the participants constantly focusing on the instruments in-front of them and trying to figure out what happens next and what to do, it serves as a distraction from all their pain and their everyday life stressors while simultaneously promoting movement, movement in which on their day-to-day life they usually don’t perform out of fear of their pain.

### Thematic analysis

**Figure 2.**
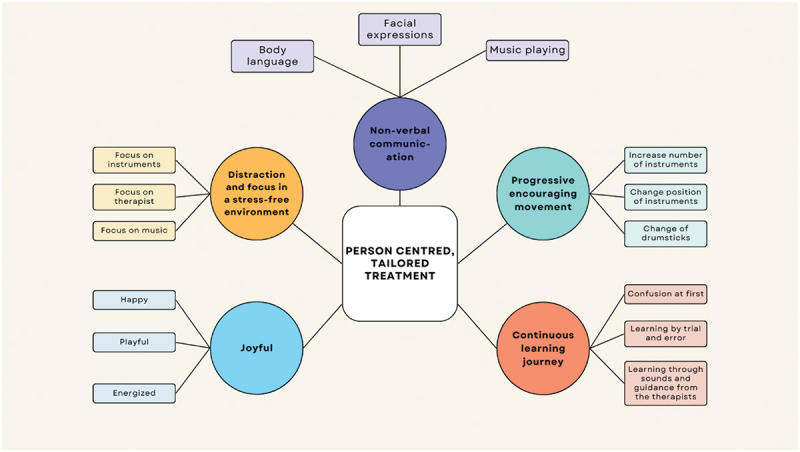
Themes and sub-themes.

The thematic analysis of the interview transcripts and video recordings led to the development of significant concepts representing the characteristics and mechanisms of FMT as a therapeutic method. These themes (Figure 2) play a crucial role in capturing the perspectives of all participants. While some aspects of the participants’ perspectives may overlap across themes, this convergence should be seen as a valid representation of their collective understandings and attitudes. Understandings and attitudes are interconnected rather than isolated concepts, highlighting the richness and complexity of the participant viewpoints. Five themes have emerged from, all branching out from the central core theme, as follows:

### Person-centred, tailored intervention

The main theme, a person-centred tailored intervention, is defined by the specific concepts and patterns identified across the entirety of the data that emphasize individualized and personalized approaches to the treatment method based on each participant individually and covers the essence of the treatment method.

In the interviews the participants described experiences of feelings of joy and happiness during the FMT sessions. Showing minimal to no signs of pain during the sessions in the video recordings as well. There was a variation in the learning process when it comes to understanding how the FMT sessions work. Overall, as the participants participated in more sessions, the more they understood the process and the better they were able to move, perform and enjoy themselves in a non-verbal manner where the only communication was body language, and the set played by the therapists and the participants together. The therapists tailor the sessions based on each participant’s individual needs, based on their pain condition and observing how well they understand the sets and adjusting sets as necessary based on the participant’s comfort and progress of learning and understanding the different sets and different movements.

### Non-verbal communication

It involves using body language, facial expressions, and music as a means of communication. It aims to provide a quiet, environment for the participants. From the video recordings it’s observed by the research team that throughout the sessions neither the therapist nor the participants speak. The therapist would start positioning the drums and cymbals for the set and as they hand the drumsticks to the participants, we researchers see how they’d lead the participant’s hand towards the drum or cymbal in which they should start playing on. In situations where the participant is confused or unsure on what to do, they maintain eye contact with the therapist to look for guidance. That’s when the therapist would try to direct them using head nods for example. Another way, as the participant stops playing, the therapist stops playing as well and will only continue playing if the participant continues playing and hits the right order planned by the therapist.

It was observed by the researchers that non-verbal communication felt new, confusing, or even strange for some participants initially. However, it often led to joy as they’re observed laughing in the video recordings. Participants also stated how it is fun and joyful in the interviews. By eliminating instructions, rules, and judgement, the non-verbal approach created a relaxed environment where participants could focus solely on the instruments and how to move or play for each set.

— We don’t talk about anything. Least possible. But you look at her facial expression and so on…And then she just points to it. It feels great! And it feels great to be with her—Participant #11

The therapists describe non-verbal communication as providing an environment where the participants can think for themselves as they want to allow the participants think about what happens now? Why are we doing this specific set now? How should I start? The therapists want to promote cognitive thinking while simultaneously putting the participants in an environment where they don’t have to think about anything else besides the instruments in front of them. The therapists want the participant to focus on how they should play and how they should move, leaving all their pain, negative thoughts, and life stressors outside the door before entering room.

— We don’t talk about how to do it, but you have to figure this out yourself. And we have an observation point which is model logic. And it must be logical. So therefore, I can’t set up a bunch of drums and the patient doesn’t know. And I don’t want to talk about it. Because then it has been lost. And this very thing of being able to figure out for yourself how it should be. To also solve one. It was someone who said this. It’s like a computer game. You must try your hand a bit. It didn’t work there, and it didn’t work there but it worked here. And then it’s that I play and answer it. — Therapist #C

This FMT method focuses on utilizing non-verbal cues to enhance communication skills through music and the body as well as to provide a stress-free, quiet, non-judgemental environment and improve overall well-being. As a non-judgemental environment fosters feelings of acceptance, safety, and emotional well-being and by eliminating stress triggers such as noise, clutter, or distractions, individuals can experience a sense of calm and relaxation. Such an environment offers support by reducing stress levels and promoting relaxation. It encourages self-reflection, mindfulness, and emotional healing.

### Progressive encouraging movement

In FMT there is a constant increase in movement during the sessions. The therapists enable that by constantly adding or remove instruments (drums and cymbals or other instruments) throughout the session. The change can also be the repositioning of the instruments to encouraging more reach movement as well as body core and rotational movement. Another way to achieve a change is by giving participants different types of drumsticks with each set to focus also on the grip strength and different hand movement. These approaches gradually increase movement with each set while simultaneously stimulating and encouraging participants to move more as the session progresses. This is achieved by strategically positioning the drums and cymbals to promote body movements tailored to the participants’ underlying pain conditions and the movements they need most. For example, participants who struggle with reach movements, drums and cymbals would be placed further away to encourage leaning forward. Another example is lateral rotations where the instruments are put to the side and further behind the participant to promote lateral rotations. At the same time, drumsticks are changed, promoting different movements and grips with the hands when using different types (e.g., short-armed drumsticks vs long-armed, thick vs thin, heavy vs light).

— Without music then you wouldn’t do these movements. To just sit like that. No, but the music gets you carried away. I couldn’t imagine sitting and holding on like that and drumming with my arms and legs if there wasn’t music. Because that would feel pretty ridiculous. — Participant #8

— I just see that now this patient needs to work on this. And then I may have written in the journal that we did this last. Next time I’ll try this maybe. There may not be an opportunity, but if I see that it works, then I will present another position. For example, we have now worked with, for example, trunk rotation. From left to right and expanded it with several instruments. Then I remove it and then I think, now we’re going to work with cross movement. — Therapist #C

The combination of the music played, and the constant change of the set contribute to the progressive encouraging movement which characterizes FMT

### Continuous learning journey

Participants who just started FMT show signs of confusion and uncertainty as seen in the videos as well as described by them in the interviews. This is due to not knowing exactly what is expected of them. The participants try out different moves until they figure out the wanted movement by trial and error as the therapist stops playing on the piano when the wanted movement isn’t achieved and continues playing if it is. Simultaneously, the therapist maintains eye-contact with the participants to provide more guidance through facial expressions and nodding as a way to confirm to the participants that they’re on the right track. Alternatively, if the participant struggles too much with a certain set, the therapist changes the set to a simpler one which is easier to follow. That helps new participants get more in touch with how the sessions go and focus more on the basics and simple sets before trying to go to more complex ones.

— She is the one who tells me, although she doesn’t tell me because she doesn’t talk to me. but she just shows with the sound how I should do it. — Participant #5

— I make a seventh chord and wait and look in one direction at the instruments. There she goes and then she turns. I confirm that then. That’s exactly what I’m looking for. I don’t say anything, I just look at the instruments. — Therapist #B

Similarly, when participants show a clear understanding of what to do and improve quickly, the therapist changes the set to a more complex one as they see that the participant is now ready to take it a step further and learn more complex sets.

### Joyful

The participants’ show joyfulness during the sessions, as they are seen laughing and smiling throughout the sessions and show a playful demeanour. The participants confirm their joy and the fun of FMT in the interviews as well. During the sessions, all negative emotions are said to be left out as stated by the participants. FMT provides the participants with the ability to focus on the music, movement and having fun. Participants can be seen laughing in the videos when they’re confused, don’t know what to do or forget the right order to follow. The therapists smile and laugh with the participants to confirm to them that the most important factor is to have fun while combining movement and music for a joyful experience that helps them exercise their movement while being happy.

— A lot of it is also that it should, that it’s good, that it feels good between me and the therapist so that you feel that it’s okay that you might miss or back down because it hurts or if you play crazy, then you can laugh, — Participant #16

— I think it’s a shame that it ended now. I would like to go on and on. because she’s so, ah, positive and stuff. And that, that’s what I need. — Participant #18

— You really get joy. There are those who start crying with you and not because they are sad, but suddenly you start touching something that you haven’t done. — Therapist #B

During the sessions, the participants are observed to be relaxed, which they’ve confirmed as well in the interviews. They’re sitting in comfort and describe it as good and even energetic.

### Distraction and focus in a stress-free environment

During the session, participants are kept active and occupied with the constant change of the set (drums, cymbals, drumsticks) as observed by the researchers through the videos. The aim is to keep the participants’ focus on the instruments and the music. The purpose of that is to distract the participants from their pain and any other life stressors. The constant change happens every 30 seconds to a minute with different sets and positions of the drums and cymbals and a change of the used drumstick which makes the participants constantly observing everything that is changing and trying to figure out what happens and what to do next. Furthermore, the participants focus as well on the therapists and what they’re doing and changing as well as focus on the music being played in order to coordinate with the therapists how to play and when to play by focusing their attention to what’s changing in the set and how to proceed.

— Sometimes you get one drumstick and sometimes two. And then they became that one (double-handed), well maybe hit in a certain way, oh now she’s not playing, oh what’s going on here now? Should I hit with both or?—Participant #14

— I change, I do new things. Something happens all the time. You never have time to think. But you always must follow along and control movements and wait. — Therapist #A

During the sessions, creating a stress-free environment is a crucial part to facilitate healing and relaxation. Carefully curated music choices are made to induce relaxation and reduce stress among participants. Music that is simple and easy to follow from a participant perspective. Which also allows the therapist to focus on the participants’ movement and notice any changes that need to be made to improve the comfort of the participant. The therapists are always welcoming and provide an inviting environment for the participants, free from distractions.

— I get relaxed and when I go into the room I really shut off everything the outside world is called if you say. Then you are like in that bubble in that room. Then that’s all that matters. And it actually gives a very pleasant feeling. That’s how I experience it. And then it turns out that it will be good for both the soul and the body. — Participant #17

— It’s always about what this patient needs. Work with parallelism directly. Yes, then we start there. Maybe start with the trunk rotation today. Maybe it fits better today. That’s how you see what a patient looks like when they come in. I slept very badly today. And then you think, then we start quietly here. —Therapist #A

The focus on the participants’ comfort (i.e., their choice of chair, keeping their shoes on or off, standing or sitting …) making them feel welcome and playing music together instead of for them, is what provides the participants with a stress-free environment where they can just relax, play music and move with zero judgement.

## Discussion

The research team identified 5 themes branching from the main theme of person-centred tailored treatment. Those themes covered the meaning of FMT throughout the interviews, video observation and re-enacting 3 treatment sessions for a deeper insight and understanding. These themes developed the meaning of FMT and the possible impact FMT has on individuals’ physical, mental and emotional well-being. Generally, it’s observed by the research team how the setting and starting steps of the treatment session is in line with the original FMT method developed by Hjelm (Hjelm, 2005; Jonsson, 2014; Paulander, 2011).

It’s observed by the researchers how the FMT treatment has an interactive approach. With different perspectives described by the therapists and participants. As from the therapist’s perspective, the aim is to conduct a session with the most amount of comfort possible for the participant. While simultaneously organize the codes and setting of the instruments in accordance with the participant’s pain condition. Keeping in mind what movements would benefit the participants the most and improve their range of movement. On the other hand, the participants see the treatment as a way to relax and just leave all their worries and stressors outside. Where their focus is what to do next with every new set and not what movements are/should be the focus as the therapists are doing. FMT uses body language and movements as ways to communicate pain like Cognitive-Behavioural Music therapy (Trondalen & Ole Bonde, 2012). While different from other music therapies as Guided Imagery and Music therapy where specifically sequenced classical music programmes are used to stimulate and sustain a dynamic unfolding of inner experiences (Trondalen & Ole Bonde, 2012). FMT on the other hand aims to avoid unfolding any inner experiences by utilizing music made to avoid any associations that could impact the participant’s mood or state of mind (Jonsson, 2014). FMT holds promise for people suffering from long-term musculoskeletal pain as a treatment method that promotes positive effects and well-being. Which is seen in previous studies on music and musical activity and their relation to emotions. Where negative emotions are reduced while positive emotions are increased (Juslin, 2019; Tervaniemi et al., 2021). The sessions exhibit a joyful demeanour. Where the participants and the therapists are united in laughter. To our current knowledge FMT has shown to be risk and harm free.

When it comes to the communication aspect of the treatment, FMT differs greatly from other music therapies such as Analytically Oriented Music therapy. Where the session starts with a verbal dialogue between the therapist and the participant. The dialogue can include roles formed beforehand and discussions as well about the music and its meaning (Trondalen & Ole Bonde, 2012). FMT on the other hand has no dialogue at all. The non-verbal communication theme explores how the session is completely non-verbal, focusing fully on body language, movement and facial expressions. From the interviews, it was noticed by the research team that the participants love the non-verbal aspect of FMT. The participants believe it’s the best approach for this treatment, as that way they don’t have to think or worry about anything at all with connects to the distraction and focus in a stress-free environment theme. Participants went further to explain how in their usual primary care it’s a stressful part to have to constantly talk about their pain condition and remember details from previous appointments when going to their new ones. The idea of just being able to show up, sit and play with nothing being said puts them at ease. Previous studies (Johansson & Lööf, 2019; Mehling et al., 2009) have reported unilateral body awareness in people with long term musculoskeletal pain. Negatively toned body awareness occurs when attention is primarily on the symptomatic and painful body. A lot of attention and time to dwell on the symptoms and pain in solitude, is shown to be negative for the health and wellbeing (Steihaug et al., 2001). The theme distraction and focus on a stress-free environment shows that the participants must pay full attention to the FMT-room -to the instruments and the music, and thereby the distraction from one´s attention on bodily symptoms is toned down. During the session, the participants can mainly focus on the active self, and the capable body. Since the therapist supports the participants in this FMT-process allowing the participant to enjoy the process of playing music as described in the joyful them. Furthermore, the research team observed from the videos how the therapist continuous change of sets encourages more movement. Adding more drums to promote more movements as is the purpose of the progressive encouraging movement theme. Finally, the progressive movement theme connects with the continuous learning theme, as participants start with simple one drum sets before moving to more advanced set. The therapists take it slow and observes participants and their progress before introducing more drums and cymbals. This is done to ensure they’ve learned enough before taking the treatment a step further to more advanced sets with more movements.

It is important to establish “‘trustworthiness’” and “‘authenticity’” in qualitative research that are like the terms validity and reliability in quantitative research. The five standards (objectivity, dependability, credibility, transferability, and application) were incorporated in this research by minimizing personal biases and subjective influences through reflecting on own biases and maintaining a reflexive journal and engaging in discussions with each other to provide diverse perspectives. The research team kept records of the research process, including decisions made, data collection methods, and analysis techniques as well as using multiple data sources (data triangulation) and methods to verify findings. Additionally, the research team reviewed and validated the findings and interpretations by spending adequate time interviewing to build rapport with the participants and therapists and gain a deep understanding of the context. Furthermore, providing rich, detailed descriptions of the research context, participants, and findings to enable readers to determine applicability and transferability by relating findings to any possible existing literature and similar studies to highlight potential parallels. Lastly, clearly articulating how findings can inform policy, practice, or further research directions and involving relevant stakeholders in the research process to ensure that findings are relevant and applicable to their needs (Lincoln & Guba, 1985; Miles et al., 2014). While this study has provided valuable insights, it is essential to acknowledge its limitations. Participants were not involved in preparing the study design nor interview guides. The reasoning being the focus of the study is the method itself. Wanting to capture the natural and spontaneous answers from all participants. With the population of long-term musculoskeletal pain being large and diverse, there is a need to study the FMT treatment and its effects on all types of long-term musculoskeletal pain causes. However, this is a long-term goal and outside our resources at the time being. However, this study has its strengths with the diverse research team and their different fields, experiences, and perspectives. This study minimizes researcher bias through structured data collection and analysis. The study’s findings have real-world implications and can be used to address the problems and suffering the target group have to deal with on a daily basis.

*In conclusion*, this research has highlighted the interpretation and description of FMT in the treatment of long-term musculoskeletal pain. By engaging multiple data sources throughout the research process, we have ensured that our findings are robust. Our results show that FMT can be a possible treatment method for long-term musculoskeletal pain conditions, as patients reported positive experiences and, to our knowledge, no harm or risks have been identified so far. The implications of these findings are significant for long-term musculoskeletal pain patients and their health care providers. Future research and testing are necessary to evaluate the effects and any possible risks in order to evaluate FMT thoroughly and throughout.

### Clinical implications

Ultimately, we encourage health care providers to actively engage with these findings to drive meaningful support and enhance the recovery process for long-term musculoskeletal pain patients. A further step in researching this method would be to further define it by conducting focus groups with both the therapists providing the FMT treatment which addresses body awareness and the participants undergoing the treatment. As the ability to pay attention to changes in the body’s signals and attention to ill health (Mehling et al., 2009), improve the quality of life by reducing the focus on the pain. As FMT has the potential to be a treatment for the long-term musculoskeletal pain population, which has no risks, no harms as up until now. FMT doesn’t involve nor include anything pharmaceutical or invasive as well.

Future research should explore the effects of FMT on the participants’ day-to-day life and its possible long-term effects. Exploring the possible differences FMT has on different causes of long-term musculoskeletal pain. Studying the possible different effects FMT has based on how long patients had pain. The research team has an ongoing project researching FMT and its effects and any possible risks. Future papers will report and focus on FMT’s effectiveness and safety. As we move forward, let us strive to apply these insights to create a more effective and inclusive treatment method for patients with long-term pain conditions.

## Appendix. Interview guides

**Table AI.** Participants interview guide.

When I say music, what do you think of? Do you think FMT fits into that (music therapy)? (how, why, why not)? How would you describe the FMT treatment you received the other day? What happens in the room from the moment you step through the door? What happens during an FMT session? What does the therapist do? What are you doing? How do you know what to do? How do you know you’re doing the right thing? Does the therapy change during a session? How? Does it hurt during the treatment? How do you communicate that to the therapist? What is the best thing about FMT? Are there more things that are good about FMT? What’s the worst thing about FMT? What role does the music play during the session? Is there anything in the FMT treatment that you don’t like at all or want to change? If yes, what?

**Table AII.** Music therapists interview guide.

When I say FMT, what do you think of? What does an FMT treatment look like? How is it done and with what tools? How are the piano pieces selected? Are all codes the same? How can the codes be described in terms of melody, rhythm and sound? Is there any progress in the codes? What does the logic look like and what governs the choices to go from one code to another? How do you choose which sets to use? (choice of percussion, positioning, seating surface, etc.) What function does the movement fulfil in the treatment? How do you know which movements to stimulate? Is there any progress in the movements? What does the logic look like and what governs the choices to go from one movement to another? What does the interaction with the patient look like? When does the FMT therapist know when it is time for an end signal? Based on your experience, what are the important mechanisms of action in FMT for people with long-term pain? In what way is the treatment adapted based on the individual patient? On the website for the FMT method1, it appears that: FMT is an individually tailored form of music therapy that stimulates the body’s development. How would you describe this in relation to patients with long-term pain? The FMT method can be a tool to stimulate people’s resources regardless of where they are in their development. In what way do you think it happens? How does your FMT treatment compare to other types of music therapy? Similarities and differences? How do you document the progression from time to time? (Written, video …) How is the documentation used? (reflection, in collaboration with the participants…)

## References

[cit0001] Ambrose, K. R., & Golightly, Y. M. (2015). Physical exercise as non-pharmacological treatment of chronic pain: Why and when. *Best Practice & Research Clinical Rheumatology*, 29(1), 120–17. 10.1016/j.berh.2015.04.022 26267006 PMC4534717

[cit0002] Biguet, G., Nilsson Wikmar, L., Bullington, J., Flink, B., & Löfgren, M. (2016). Meanings of “acceptance” for patients with long-term pain when starting rehabilitation. *Disability and Rehabilitation*, 38(13), 1257–1267. 10.3109/09638288.2015.1076529 26305503

[cit0003] Bradshaw, C., Atkinson, S., & Doody, O. (2017). Employing a qualitative description approach in health care research. *Global Qualitative Nursing Research*, 4, 2333393617742282. Retrieved November 24, 2017, from 10.1177/2333393617742282 29204457 PMC5703087

[cit0004] Braun, V., & Clarke, V. (2006). Using thematic analysis in psychology. *Qualitative Research in Psychology*, 3(2), 77–101. 10.1191/1478088706qp063oa

[cit0005] Braun, V., & Clarke, V. (2014). What can “thematic analysis” offer health and wellbeing researchers? *QHW*, 9, 26152. 10.3402/qhw.v9.26152 25326092 PMC4201665

[cit0006] Carter, N., Bryant-Lukosius, D., DiCenso, A., Blythe, J., & Neville, A. J. (2014). The use of triangulation in qualitative research. *Oncology Nursing Forum*, 41(5), 545–547. 10.1188/14.ONF.545-547 25158659

[cit0007] Edwards, J. (Ed.). (2015). *The Oxford handbook of music therapy*. Oxford University Press. 10.1093/oxfordhb/9780199639755.001.0001

[cit0008] El-Tallawy, S. N., Nalamasu, R., Salem, G. I., LeQuang, J. A. K., Pergolizzi, J. V., & Christo, P. J. (2021). Management of musculoskeletal pain: An update with emphasis on chronic musculoskeletal pain. *Pain Ther*, 10(1), 181–209. 10.1007/s40122-021-00235-2 33575952 PMC8119532

[cit0009] Fancourt, D., & Finn, S. (2019). *What is the evidence on the role of the arts in improving health and well-being? A scoping review*. WHO Regional Office for Europe https://www.ncbi.nlm.nih.gov/books/NBK553773/32091683

[cit0010] FMT-behandlingscentrum (u.å.). (n.d.). *Musikterapi FMT-modellen* - informationsbroschyr. På uppdrag av Region Sörmland

[cit0011] Garza-Villarreal, E. A., Pando, V., Vuust, P., & Parsons, C. (2017). Music-induced analgesia in chronic pain conditions: A systematic review and meta-analysis. *Pain Physician*, 20(7), 597–610. 10.36076/ppj/2017.7.597 29149141

[cit0012] Hjelm, L. (2005). *Med musik som medel. FMT-metoden. Som Den Blev Till Falun Scanbook AB*. Musikterapiinstitutet.

[cit0013] Johansson, U.-B., & Lööf, H. (2019). A body in transformation - an empirical phenomenological study about fear-avoidance beliefs towards physical activity among persons experiencing moderate to severe rheumatic pain. *Journal of Clinical Nursing*, 28(1–2), 321–329. 10.1111/jocn.14606 29971848 PMC8045552

[cit0014] Jonsson, A. S. (2014). Functionally oriented Music Therapy (FMT) as a method of improving children’s ability to function at school. Master’s thesis. *DIVA*. 2014. Ingesund College of Music. https://www.ncbi.nlm.nih.gov/books/NBK553773/

[cit0015] Juslin, P. N. (2019). *Musical Emotions Explained*. Oxford University Press. 10.1093/oso/9780198753421.001.0001

[cit0016] Lincoln, Y. S., & Guba, E. G. (1985). *Naturalistic inquiry*. Sage Publications, Inc.

[cit0017] Mason, J. (2002). *Qualitative researching* (2nd ed.). SAGE.

[cit0018] Mcleod, S. (2009). Piaget’s theory and stages of cognitive development. *Simply Psychology*. 10.5281/zenodo.15241970

[cit0019] Mehling, W. E., Gopisetty, V., Daubenmier, J., Price, C. J., Hecht, F. M., Stewart, A., & García, A. V. (2009). Body awareness: Construct and self-report measures. *PLOS ONE*, 4(5), e5614. 10.1371/journal.pone.0005614 19440300 PMC2680990

[cit0020] Miles, M. B., Huberman, A. M., & Saldana, J. (2014). *Qualitative data analysis: A methods sourcebook*. Sage.

[cit0021] Mingers, J. (2010). The contribution of systemic thought to critical realism. *JCR*, 10(3), 303–330. 10.1558/jcr.v10i3.303

[cit0022] Okolo, A., Olorunsogo, T., & Babawarun, O. (2024). None Tolulope Olorunsogo, none Oloruntoba Babawarun. Cultural variability in pain perception: A review of cross-cultural studies. *IJSRA*, 11(1), 2550–2556. 10.30574/ijsra.2024.11.1.0339

[cit0023] Palinkas, L. A., Horwitz, S. M., Green, C. A., Wisdom, J. P., Duan, N., & Hoagwood, K. (2015). Purposeful sampling for qualitative data collection and analysis in mixed method implementation research. *Adm Policy Ment Health*, 42(5), 533–544. 10.1007/s10488-013-0528-y 24193818 PMC4012002

[cit0024] Paulander, A. S. (2011). The Meaning of Music Therapy: A phenomenological study of participant’s experiences. *Diva* https://www.ncbi.nlm.nih.gov/books/NBK553773/

[cit0025] Raja, S. N., Carr, D. B., Cohen, M., Finnerup, N. B., Flor, H., Gibson, S., Keefe, F. J., Mogil, J. S., Ringkamp, M., Sluka, K. A., Song, X.-J., Stevens, B., Sullivan, M. D., Tutelman, P. R., Ushida, T., & Vader, K. (2020). The revised International Association for the Study of Pain definition of pain: Concepts, challenges, and compromises. *Pain*, 161(9), 1976–1982. 10.1097/j.pain.0000000000001939 32694387 PMC7680716

[cit0026] Rosin, A., Ericsson, M., & Larsson, K. (2015). The effects of functionally oriented music therapy on body function and quality of life in chronic stroke survivors and on patients with Parkinson’s disease. *Music Medicin*, 7(2), 14–19.

[cit0027] Söderlund, A., & Asenlöf, P. (2010). The mediating role of self-efficacy expectations and fear of movement and (re)injury beliefs in two samples of acute pain. *Disability and Rehabilitation*, 32(25), 2118–2126. 10.3109/09638288.2010.483036 20443673

[cit0028] Statens beredning för medicinsk och social utvärdering. (2010). Rehabilitering vid långvarig smärta. *En Systematisk Litteraturöversikt*. SBU-rapport nr 198. https://www.sbu.se/341

[cit0029] Steihaug, S., Ahlsen, B., & Malterud, K. (2001). From exercise and education to movement and interaction. Treatment groups in primary care for women with chronic muscular pain. *Scandinavian Journal of Primary Health Care*, 19(4), 249–254. 10.1080/02813430152706783 11822650

[cit0030] Syed, J., Mingers, J., & Murray, P. (2010). Beyond rigour and relevance: A critical realist approach to business education. *Manage Learning*, 41(1), 71–85. 10.1177/1350507609350839

[cit0031] Tervaniemi, M., Makkonen, T., & Nie, P. (2021). Psychological and physiological signatures of music listening in different listening environments-an exploratory study. *Brain Sci*, 11(5), 593. Retrieved May 3, 2021. 10.3390/brainsci11050593 34063693 PMC8147775

[cit0032] Treede, R. D., Rief, W., Barke, A., Aziz, Q., Bennett, M., Benoliel, R., Cohen, M., Evers, S., Finnerup, N., First, M., Giamberardino, M. A., Kaasa, S., Korwisi, B., Kosek, E., Lavand’homme, P., Nicholas, M., Perrot, S., Scholz, J., Schug, S., … Wang, S.-J. (2019). Chronic pain as a symptom or a disease: The IASP classification of chronic pain for the international classification of diseases (ICD-11). *Pain*, 160(1), 19–27. 10.1097/j.pain.0000000000001384 30586067

[cit0033] Trondalen, G., & Ole Bonde, L. (2012). *Music Therapy: Models and Interventions. Music, Health, and Wellbeing*. Advance online publication., 41–62. 10.1093/acprof:oso/9780199586974.003.0004

[cit0034] Turk, D. C., Okifuji, A., Sinclair, J. D., & Starz, T. W. (1996). Pain, disability, and physical functioning in subgroups of patients with fibromyalgia. *Journal of Rheumatology*, 23(7), 1255–1262. 8823701

[cit0035] Williams, L., Rycroft-Malone, J., & Burton, C. R. (2017). Bringing critical realism to nursing practice: Roy Bhaskar’s contribution. *Nursing Philosophy*, 18(2), 10.1111/nup.12130 27381640

